# Houston Methodist Ruptured Abdominal Aortic Aneurysm Guidelines

**DOI:** 10.14797/mdcvj.1177

**Published:** 2023-03-07

**Authors:** Vy C. Dang, Peter J. Osztrogonacz, Paul Haddad, Shashank Sharma, Stuart J. Corr, Maham Rahimi

**Affiliations:** 1 Texas A&M School of Medicine, Bryan, Texas, US; 2 Houston Methodist Hospital, Houston, Texas, US; 3 Rice University, Houston, Texas, US; 4 Swansea University Medical School, Swansea, United Kingdom; 5 Weill Cornell Medical College, New York, New York, US

**Keywords:** ruptured abdominal aortic aneurysm, transfer of care, RAAA Timeout, patient outcomes, protocol, guidelines, acute aortic syndromes, THROMBINS2

## Abstract

Ruptured abdominal aortic aneurysm (RAAA) is an acute aortic condition that requires emergent intervention and appropriate continuity of care to optimize patient outcomes. We describe the standardized RAAA protocol at the Houston Methodist Hospital Acute Aortic Treatment Center, developed to navigate critical patient transfer periods safely and efficiently, make crucial decisions about surgical intervention, and clearly communicate these plans with other care team providers. Our workflow is organized into five phases: prehospital, preoperative, intraoperative, postoperative, and post-discharge. We identify the transfer center, anesthesia, operating room nursing staff, surgeons, and intensive care unit as key entities of our acute aortic pathology care team. This systematic protocol for the management of acute aortic emergencies such as RAAA identifies critical decision points, potential complications at each stage, and recommendations for best practice.

## Introduction

Ruptured abdominal aortic aneurysm (RAAA) is an acute aortic condition that requires immediate action and appropriate continuity of care to optimize patient outcomes. The decline in the number of RAAAs in the United States (US), along with the innate fast-paced nature of aortic emergencies, has left fewer opportunities for the medical team—emergency room personnel, nurses, anesthesiologists, fellows, residents, surgeons, and critical care physicians—to improve and maintain their skillset for RAAA management.^1^ Consequently, a systematic standardized protocol-driven approach is essential to improve the management of aortic emergencies as well as perioperative morbidity, mortality, and long-term survival. Although current Society of Vascular Surgery (SVS) Guidelines provide recommendations for the perioperative management of RAAA,^2^ aortic centers with lower volumes of RAAA cases require a robust, standardized protocol applicable to their specific needs.

In this article, we summarize the internal protocol of the Houston Methodist Hospital (HMH) Acute Aortic Treatment Center (AATC) to provide guidance to vascular surgeons in training and to low-volume aortic centers, guided by the five phases of surgical care outlined by the American College of Surgeons (ACS): preoperative, perioperative, intraoperative, postoperative, and post-discharge.

## Preoperative Phase

### Transferring Process

Before the patient arrives from a transferring hospital or emergency room, a general call should be initiated. The transferring hospital should provide the patient’s name, date of birth, medical record number, reason for transfer, code status, the presence/absence of metastatic cancer, and anticoagulants. The receiving hospital should provide instructions for transfer, including a reminder to maintain permissive hypotension and avoid intubation and sedation, if possible.

After 5 minutes, another call should take place. We recommend the development and utilization of an efficient, HIPAA-compliant alert system that reaches the surgeon, anesthesiologists, operating room (OR) staff, and intensive care unit (ICU) staff for rapid transfer of information. This alert should include the current vitals, fluids, pressors, or other drugs administered, the patient level of consciousness, whether they are actively undergoing cardiopulmonary resuscitation, patient code status, and the patient transfer destination and time frame.^3^ The alert system may be cloud-based and allow for sharing of computerized tomography angiogram (CTA) images from the initial diagnostic workup. At the HMH AATC, we use Life Image® and are transitioning to Ambra®, a cloud-based medical image management system.

The hybrid OR should be prepared for patient transfer and operation. All patients with RAAA accepted for transfer should be transferred directly to the hybrid OR. A blood type and screen should be performed in the receiving hospital, if not already done, but should not delay transfer. The transferring hospital should be instructed to avoid intubation and sedation, if possible. Intravenous (IV) access should be obtained via two large-bore peripheral lines.^2,3^ Fluid resuscitation introduces a risk of coagulopathy, hypothermia, and acidosis and thus should be avoided if the patient is conscious. If necessary, the patient may receive balanced transfusion (1:1:1 packed red blood cells:fresh frozen plasma:platelets).

If the patient requires resuscitation, the goal is to achieve permissive hypotension, defined as maintaining patient consciousness and systolic blood pressure (SBP) between 70 mm Hg and 90 mm Hg through the conservative delivery of IV crystalloids/colloids to prevent major blood loss during the operation.^3^ Permissive hypotension and controlled hypotension, which is the lowering of blood pressure with vasodilators and/or beta blockers, are two approaches to maintaining SBP between 50 mm Hg and 100 mm Hg and achieving hypotensive hemostasis.^4^ However, because of patient variability and the risks of prolonged and severe hypotension (ie, mesenteric ischemia, acute kidney injury), we prefer to use permissive hypotension as our guide in resuscitation at the HMH AATC.

### Remote Patient Evaluation by Surgeon

**Figure 1:**
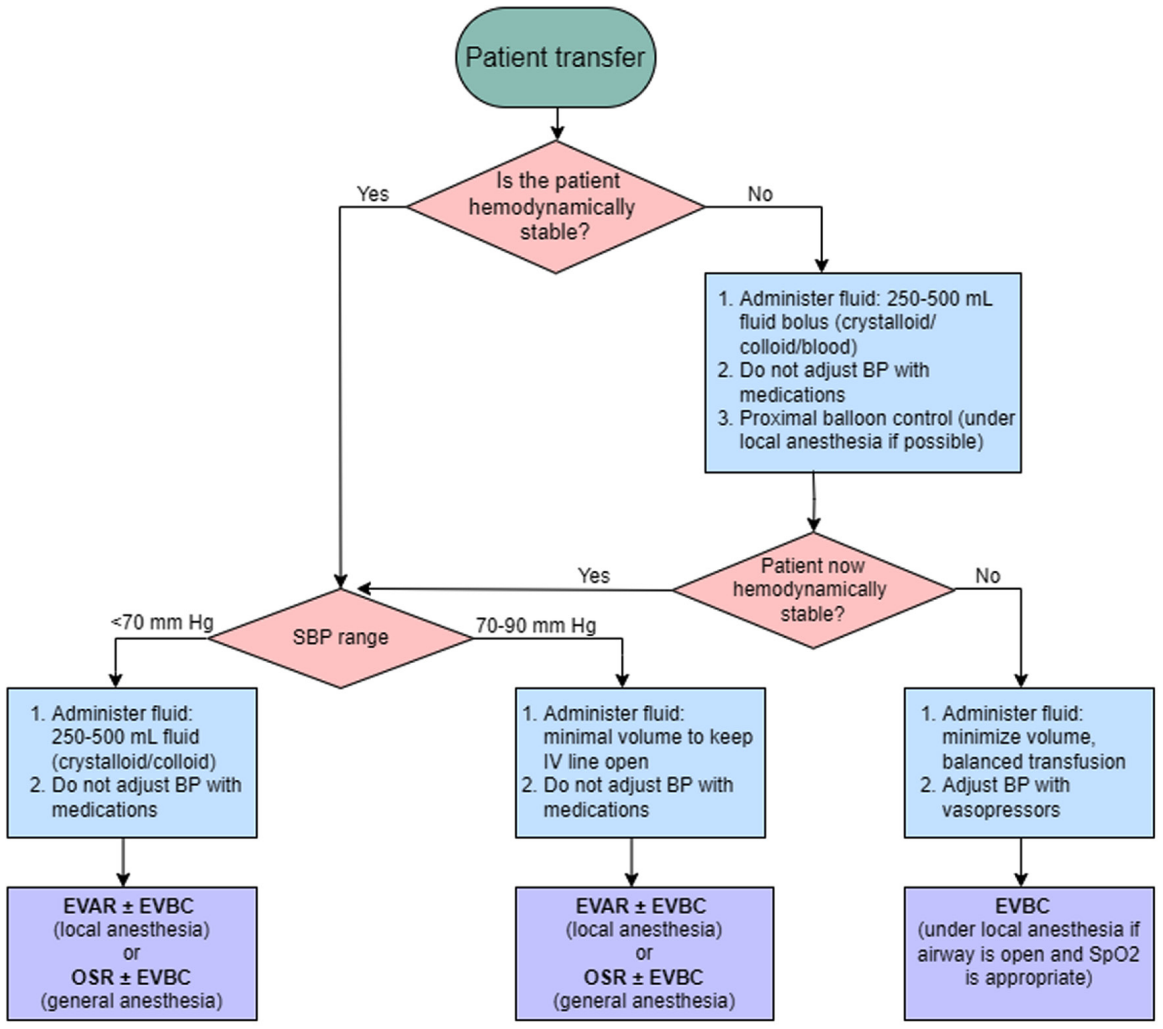
Algorithm for preoperative blood pressure and volume management. Hemodynamic stability is defined as patient consciousness with no fluid/pressors to maintain blood pressure between 70-90 mm Hg for at least 5 minutes. BP: blood pressure; EVAR: endovascular aortic repair; EVBC: endovascular balloon control; OSR: open surgical repair; SBP: systolic blood pressure.

The surgeon must first determine whether proximal control is necessary (Figure 1) and what method is optimal, be it percutaneous endovascular balloon control (EVBC) or aortic cross-clamping through an abdominal midline incision. EVBC is performed under local anesthesia and recommended for hemodynamically unstable patients and/or those unresponsive to fluids and drugs.^5,6,7,8^ Hemodynamic stability is defined in these guidelines as consciousness with no fluid or pressors to maintain blood pressure between 70 mm Hg and 90 mm Hg for at least 5 minutes. Additionally, supraceliac aorta free of aneurysmal or major atherosclerotic disease and an adequate length of healthy aortic segment is necessary to safely perform balloon control. EVBC can be followed by either endovascular aortic repair (EVAR) or open surgical repair (OSR), while aortic cross-clamping necessitates OSR.

The surgeon should have approximately 30 minutes during the transfer period to plan the procedure using 3-dimensional modeling software, such as Siemens Syngo.via®, Osirix™, 3Mensio Vascular®, or TeraRecon, to evaluate whether the patient will undergo EVAR or OSR.^9^ SVS Guidelines recommend EVAR whenever patient anatomy allows for it.^2^ CTA findings indicating the utility of EVAR include sufficient diameters of femoral and iliac arteries without excessive tortuosity or calcifications, an aortic bifurcation diameter of at least 17 mm, and sufficient diameter, angle, and length of the aneurysmal neck (≥ 15 mm) (Table 1).^2,4^ Inability to perform EVAR due to access-related issues can be remedied with either open exposure of the common femoral artery or iliac conduits. If EVAR is appropriate, the radiology technician should be alerted.

**Table 1:** Important computerized tomography angiogram findings prior to treatment of RAAA.^2,4^ EVAR: endovascular aortic repair; OSR: open surgical repair; AAA: abdominal aortic aneurysm; RAAA: ruptured abdominal aortic aneurysm


EVAR	OSR	OTHER CONSIDERATIONS REGARDLESS OF EVAR OR OSR

Diameter (< 32 mm), angle (< 60°) and length (≥ 10 mm depending on type of endograft) of aneurysmal neck	Extent of healthy artery free of calcifications and aneurysms	Superior mesenteric artery: level of aortic origin, presence of stenosis/obstruction

Diameter of aortic bifurcation (≥ 17 mm)	Distal disease	

Diameter of common iliac arteries (≥ 5 mm)	Free vs contained RAAA	

Diameter of external iliac arteries (≥ 5 mm)	Presence of retro-aortic left renal vein	

Diameter of femoral arteries without excessive tortuosity or calcifications (access point)	Thoracoabdominal aneurysm, inflammatory AAA, horseshoe kidneys	


RAAA patients with aortic anatomy unsuitable for EVAR should be treated with OSR. Preoperative planning for OSR includes decision-making between transperitoneal versus retroperitoneal approaches, assessment of the proximal and distal clamping zones, the presence of retroaortic or circumaortic left renal vein, and the evaluation of visceral circulation (Table 1).^2,3,4^ Although the transperitoneal supraceliac approach is considered the gold standard exposure, an alternative retroperitoneal exposure is required for multiple recent prior abdominal surgeries, horseshoe kidney, inflammatory aneurysm, or the presence of thoracoabdominal aneurysm.

The preoperative assessment of CTA to evaluate proximal and distal clamping sites helps to avoid calcified or aneurysmal aorta and/or iliac arteries. Excessive bleeding due to left renal vein injury can be avoided by identifying the aberrant left renal vein course on the preoperative CTA, contributing to improved perioperative mortality. Finally, evaluation of the visceral circulation is considered one of the most important steps of preoperative planning to avoid postoperative mesenteric ischemia. The perioperative CTA should be evaluated carefully to assess the degree of superior mesenteric artery (SMA) disease, evidence of prior hemicolectomy (especially involving the transverse colon), absence of middle colic artery, and/or the patency of the inferior mesenteric artery (IMA). In addition to the previous CTA findings, the IMA back bleeding and Doppler evaluation of small and large bowel during OSR should guide consideration for SMA intervention versus IMA reimplantation.

The surgeon should be prepared for open surgical conversion during the EVAR procedure; thus we cannot stress enough how crucial the preoperative anatomical evaluation is in both OSR and EVAR.

## Intraoperative Phase

### RAAA Timeout

As soon as the RAAA patient arrives at the OR, the vascular surgeon and anesthesiologist should have a focused conversation with the transferring personnel regarding the patient’s hemodynamic status during transfer and the type, dose, and exact time of administered drugs, crystalloids, colloids, and blood products. Patient handoff is an especially crucial moment in the management of RAAA, as the treating medical staff must be aware of prior therapy to make an informed hemodynamic assessment. In our institution, this conversation is called “RAAA Timeout” and plays a central role in the assessment of hemodynamic status.

The RAAA Timeout begins with the vascular surgeon asking the transferring personnel the following questions:

- Was there hemodynamic instability during transfer (Figure 1)?
- Were any pressors given during transfer?
- Were any crystalloids/colloids/blood products given during transfer?

Then the surgeon assesses the patient’s consciousness level. These focused evaluations determine the necessity of EVBC. The preoperative CTA determines OSR versus EVAR. During either procedure, the surgeon should be prepared for EVBC if the patient becomes hemodynamically unstable.

#### Nursing

The OR nursing staff should prepare either an EVAR or OSR preference cart based on the preoperative planning after the type of repair is communicated clearly by the attending vascular surgeon. A standardized workflow should be followed, including placement of a Foley catheter, prepping of the patient, checking the room temperature, placement of Zoll pads, preparation of the cell saver device, and preparation of a crash cart in anticipation of a code blue scenario. If the patient is to receive general anesthesia, they should be fully prepped and draped for balloon control prior to induction. The patient should be prepped from the nipples to knees in the supine position, with the right arm tucked and left arm on an arm board.

#### Anesthesia

Resuscitation should follow the principles of permissive hypotension as detailed in the preoperative phase, and balanced transfusion is recommended. Thromboelastography, or pulse pressure variation (PPV) in mechanically ventilated patients, may replace Swan-Ganz catheters in providing information about fluid status to guide resuscitation. If PPV < 11%, the patient has received an appropriate amount of fluid, but if PPV > 11%, more fluid is required to maintain optimal end organ perfusion.^10^

Obtaining venous access, placement of an arterial line, EVBC positioning, and percutaneous EVAR procedures can be completed under local anesthesia. OSR is performed under general anesthesia, with opioid administration via (1) continuous infusion of remifentanil for more stable pain control in EVAR/shorter procedures, or (2) bolus injections of fentanyl-midazolam for OSR/longer procedures.^11^ Patient core body temperature should be kept above 36°C; failure to do so is associated with increased perioperative morbidity and mortality.^4^ Ideally, EVBC and/or EVAR should be performed under local anesthesia to mitigate the detrimental impact of general anesthesia on hemodynamics. If intubation is necessary, vasoplegia and myorelaxation should be avoided to prevent hemodynamic collapse.

Potential cardiovascular complications include a myocardial infarction (MI), which should be assessed and managed intraoperatively with a 12-lead electrocardiogram and transesophageal echocardiogram, and hypotension associated with ischemic reperfusion and unclamping of the aorta. Anesthesia should be prepared to administer fluid or pressors at the time of unclamping.

#### Surgeon

The vascular surgeon must be prepared for hemodynamic instability during patient intubation and must react accordingly, either by performing EVBC or supraceliac cross clamping. We recommend performing EVBC from the side of the straightest iliac system since, during the EVAR procedure, the cannulation of the contralateral limb is done through the straightest iliac.^4^ The balloon is ideally placed above the renal arteries if the aorta is normal and free of disease, and the time elapsed should be recorded to avoid liver and reno-visceral ischemia. In aortic cross clamping, we prefer obtaining supraceliac over infrarenal control given that the supraceliac approach is usually more readily attainable in RAAA patients. Heparin should be administered after proximal aortic control; we recommend adjusting the dose in accordance with activated clotting time.

Bleeding from the proximal anastomosis may be prevented with complete transection of the aorta for end-to-end anastomosis, which makes it possible to visualize the back wall and place repair sutures. Distal bleeding, most often associated with venous injury due to complete dissection of the posterior wall of the aortic bifurcation or iliac arteries, can be prevented by the following techniques. If anastomosis is done at the aortic bifurcation, only partial transection of the aorta is favorable to prevent venous injury. If anastomosis is done at the common iliac artery, avoiding posterior wall dissection is favorable, with only anterolateral and medial control of the artery for clamp placement to avoid venous injury. If venous injury occurs, packing and pressure often stop the bleeding since attempts to repair the vein will cause further tearing. If packing does not stop a venous bleed, an assistant should help with proximal-distal control with two sponge sticks and cell-saver suction. This can often provide appropriate visualization for venous repair. If venous injury is located posterior to any aortic or iliac dissection and the bleeding does not stop with packing and pressure, complete transection of the aorta and/or iliac arteries is important for appropriate visualization and potential repair of the venous injury.

Suprarenal or supraceliac posterior aortic bleeding due to clamp injury or right-angle injury to the lumbar or intercostal arteries is inaccessible to visualization and surgical repair. These types of injuries can be controlled by wrapping that level of the aorta with an Evarrest® Fibrin Sealant Patch circumferentially and tightly suturing the patch anteriorly to control aortic bleeding. The retracted intercostal or lumbar arteries will eventually thrombose because they are almost impossible to locate and suture-ligate. The surgeon should also take time to suture-ligate lumbar arteries prior to aortic anastomosis.

Recognizing the reasons behind anastomotic bleeding is paramount prior to repair. Anastomotic bleeding can develop in several ways: a loose suture line, development of pleat, an aortic tear, suturing a heavily calcified aortic wall without incorporation of adventitia, or coagulopathy. Loose suture lines can be addressed by pulling up on the prolene suture using a nerve hook to tighten the loose suture. This loop of prolene suture is then tied down to an adjacent, newly placed prolene suture either with a surgeon instrument tie or passing a 3-0 silk suture through the loop and performing a basic surgical knot. If the surgeon initially traveled too far apart and caused a pleat formation, a figure-eight prolene suture can fix the problem. If there is an aortic tear at the anastomotic site, it can be repaired by a figure-eight parallel to the graft, suturing only the aorta. If the anastomotic bleed is due to aortic calcification, it is most likely caused by failing to incorporate aortic adventitia. The sutures should be redone at that level, incorporating the adventitia and graft using prolene sutures. For fragile aortic tissue, we recommend pledget reinforcement for figure-eight repair sutures or an external strip of Teflon felt to incorporate into the entire anastomosis (similarly used in OSR of aortic dissection).^12^

## Postoperative Phase

### Early Complications in the ICU

Patients should be sent to the ICU postoperatively, as complications may arise in multiple organ systems and must be carefully monitored and appropriately managed as early as possible to avoid end-organ damage and/or death (Table 2).

**Table 2:** Postoperative complications of ruptured abdominal aortic aneurysm repair and treatment recommendations.^4,12,13,14,15,16,17,18,19,20,21,22,23,24^ BNP: brain natriuretic peptide; BUN: blood urea nitrogen; CBC: complete blood count; CT: computed tomography; CSF: cerebrospinal fluid; CXR: chest X-ray; ECG: electrocardiogram; ESRD: end-stage renal disease; FiO_2_: fraction of inspired oxygen; HLA: human leukocyte antigen; IAP: intra-abdominal pressure; INR: international normalized ratio; IMA: inferior mesenteric artery; IVC: inferior vena cava; LMWH: low-molecular weight heparin; MAP: mean arterial pressure; PaO_2_: arterial partial pressure of oxygen; PEEP: positive end-expiratory pressure; PCR: polymerase chain reaction; PT: prothrombin time; PTT: partial thromboplastin time; SMA: superior mesenteric artery; SSEP: somatosensory evoked potentials; SQ: subcutaneous; tcMEP: transcranial motor evoked potentials; TEG: thromboelastography; THROMBINS_2_: thienopyridines, renin-angiotensin system blockade, oxygen, morphine, beta-blockers, invasive cardiac interventions, nitroglycerin, statin/salicylate; VV ECMO: veno-venous extracorporeal membrane oxygenation


ORGAN/SYSTEM	COMPLICATION	PRESENTATION/MONITOR	PREVENTION	TREATMENT

Central nervous system	Spinal cord ischemia^4,12^	Neurological symptoms, spinal cord (neurophysiologic) monitoring: SSEP and tcMEP, CSF drain monitoring, BP, oxygenation	Avoid sustained hypotension: intraoperative MAP > 80 mm Hg	Increase MAP using IV fluid bolus up to 2 L, vasopressors, inotropes. Spinal drain, increase hemoglobin, oxygenation.

Cardiovascular	Cardiac dysfunction^4,14,15^	ECG, echocardiograms, serum troponin, serum BNP, lactate	Avoid volume overload (give furosemide post-op day 3 if necessary). Avoid tachycardia. Intraoperative communication with anesthesia during reperfusion to avoid excessive ischemic reperfusion injury.	Practice THROMBINS2

	Bleeding	CBC (hemoglobin, platelet), fibrinogen, INR/PT/PTT, calcium, TEG, BUN	Correct coagulopathy early. Address surgical bleed early. Appropriate surgical technique (avoid venous injury) prior to heparinization. Avoid hemodilution. Avoid excessive/prolonged heparinization.	If surgical bleeding, need early mobilization to OR prior to the patient becoming coagulopathic. With bleeding patient, practice balanced transfusion. For ESRD patients, give desmopressin.

	Deep venous thrombosis^4,16,17^	Lower/upper extremity swelling, D-dimer, venous ultrasound	Early mobilization, intermittent pneumatic compression, subcutaneous heparin/LMWH, removal of central access catheters, when possible. DVT prophylaxis (after successful repair and stable hemoglobin for 24 h).	Heparin/LMWH (SQ), Fondaparinux (SQ), warfarin. If unable to anticoagulate, place IVC filter.

	Access complication	Limb ischemia due to access site/limb thrombosis, groin complications (seroma, hematoma, infection), which can be associated with drainage, erythema, wound dehiscence	Hourly neurovascular assessment for signs of ischemia, daily assessment for wound complication	Recognize the access site complication and address it in a timely manner by returning to the OR.

Pulmonary	Transfusion-associated cardiogenic overload^18^	Elevated serum BNP, acute/worsening pulmonary edema, cyanosis, hypoxia with absence of other specific causes, unexplained cardiovascular changes	Slower infusion rate, preemptive diuretics	Diuretics

	Transfusion-related acute lung injury^18^	Increased oxygen demand, pink/frothy secretions from endotracheal tube, fever, hypotension, cyanosis	Donor screening/deferral for anti-HLA antibodies	Supportive treatment

	Ventilator-associated pneumonia ^4,21,22^	Development within 48 hours of endotracheal intubation. Respiratory decline, lung infiltrates (CXR), fever, cough. Blood cultures, sputum cultures, PCR testing from nasal swabs.	Elevation of head of the bed 30-45° to prevent aspiration. Oral chlorhexidine. Oral care, hospital hygiene measures (handwashing), timely vaccinations for patients and providers. Reduce number of days on mechanical ventilation.	Empiric antibiotic therapy based on institution-specific antibiogram.

	Acute respiratory distress Syndrome^4,18,19,20,23,24^	Lung injury onset within 1 week of clinical insult, with symptoms unexplained by cardiac failure or fluid overload. Bilateral, diffuse opacities (CXR/CT). Respiratory failure. Severe hypoxemia. Decreased PaO2/FiO2 ratio (≤ 300 mm Hg).	On mechanical ventilation, maintain low tidal volume (≤ 6 mL/kg predicted body weight). Limit blood transfusion. Reduce number of days on mechanical ventilation.	Noninvasive positive pressure ventilation, low plateau pressures (< 30 cm H2O), permissive hypercapnia, appropriate titration of PEEP (≥ 5 cm H2O), prone ventilation. Continuous IV infusion of neuromuscular blockers. Discuss VV ECMO.

Gastrointestinal	Abdominal compartment syndrome^4^	Difficulty ventilating patient, sustained IAP ≥ 20 mm Hg while patient is paralyzed.	Preemptive diuretics; assess for hemorrhage.	Decompression laparotomy. Diuretics, dialysis/hemofiltration, intravenous paralytic agents.

	Ischemic colitis^4^	Abdominal distension and/or pain, fevers, and early bowel movements (post-op day 0/1). Leukocytosis. Persistent acidosis. Diagnose with emergent Flex Sig.	Avoid strong vasoconstrictors (ie, vasopressin or phenylephrine). Revascularize IMA or diseased SMA.	Early recognition by early Flex Sig. Exploratory laparotomy with possible bowel resection. Supportive therapy (fluids, BP support, bowel rest, antibiotics, NG tube).

Renal	Acute kidney injury^4^	FENa > 1%, muddy brown casts in urine sediment, low urine output, anasarca.	Minimize vasopressors, avoid prolonged suprarenal aortic cross-clamping, avoid nephrotoxic drugs	Renal replacement therapies (ie, dialysis)

Musculoskeletal	Lower extremity ischemia	Physical exam and noninvasive vascular studies.	Intraoperative heparin	Therapeutic anticoagulation, thrombectomy, fasciotomies for extremity compartment syndrome.


The onset of neurological symptoms after an RAAA repair may indicate spinal cord ischemia during the thoracoabdominal aneurysm repair, which can be detected intraoperatively through neurophysiologic monitoring, somatosensory evoked potentials, and transcranial motor evoked potentials, to guide supportive treatment.^13^ However, spinal cord ischemia can be prevented intraoperatively by avoiding sustained hypotension and maintaining MAP above 80 mm Hg, in addition to postoperative monitoring of cerebral spinal fluid drain status (if one is placed), oxygen delivery (oxygen saturation and hemoglobin levels), and patient status (blood pressure, perfusion pressure, and cognitive status).^4,14^

Common cardiovascular complications include cardiac dysfunction and coagulopathies, all of which require clear communication and cooperation between the surgery and anesthesia to achieve the crucial balance between bleeding and thrombosis. Upon diagnosis of cardiac dysfunction, THROMBINS_2_ (thienopyridines, renin-angiotensin system blockade, oxygen, morphine, beta-blockers, invasive cardiac interventions, nitroglycerin, statin/salicylate) should guide the treatment plan. THROMBINS_2_ is a new approach developed by Advanced Cardiovascular Life Support that replaces MONA (morphine, oxygen, nitroglycerin, and aspirin) for the management of acute coronary syndromes.^15,16^ Bleeding should constantly be monitored and addressed early, prior to the patient becoming coagulopathic. Surgical management of bleeding is discussed in detail in the intraoperative phase. Deep venous thrombosis (DVT) is preventable with multiple techniques such as early mobilization, intermittent pneumatic compression, subcutaneous heparin and low molecular weight heparin, and removal of central access catheters, when possible.^17,18^ DVT prophylaxis should begin after a successful RAAA repair and after the patient hemoglobin has been stable for 24 hours.

Another complication associated with RAAA treatment is based on access site complications. In EVAR or OSR, the access site complication could be due to ischemia, seroma, hematoma, and/or infection. Daily assessment of groin incisions is recommended for early recognition of these complications and addressing them in a timely manner in the OR for favorable outcomes. Lower extremity ischemia after EVAR could be due to a kink in the iliac limb of the stent graft due to iliac tortuosity, small aortic bifurcation (< 17 mm), and/or poor outflow. Furthermore, lower extremity ischemia after OSR is most often due to iliac flow-limiting dissection from clamping, poor outflow due to severe atherosclerotic disease in iliac/femoral arteries, and/or iliac graft kinking. In the era of endovascular therapy, surgeons should consider an alternate plan if the patient returns with proximal anastomosis suture line disruption with bleeding that requires endovascular salvage. The possibility of the patient returning with a suture line disruption guides the surgeon’s modification of the graft during RAAA OSR, as the aortic portion of the OSR graft must fit the stent graft used in endovascular salvage. It is important, however, to not make the aortic portion of the graft too long during OSR because it may lead to iliac kinking, resulting in limb thrombosis. Lower extremity ischemia can be assessed based on physical exam and noninvasive vascular studies (ie, Doppler ultrasound) and prevented with intraoperative heparin. If detected, the patient may begin therapeutic anticoagulation, undergo thrombectomy, or require fasciotomies if extremity compartment syndrome develops. Other access site complications might be due to seroma, hematoma, and/or infection in the groin. Patients with active seroma drainage should be taken back to the OR for re-exploration of the groin with possible muscle flap creation, SPY limb angiography, and/or lymphatic ligation. Most often, hematoma complications can be managed nonoperatively if they are not associated with drainage, skin compromise, and/or expanding hematoma. The groin infection usually is the late sequelae of seroma and hematoma formation and requires surgical intervention.

Pulmonary complications include transfusion-associated cardiogenic overload (TACO), transfusion-related acute lung injury (TRALI), acute respiratory distress syndrome (ARDS), and ventilator-associated pneumonia (VAP).^4,19,20,21^ TACO is improved with diuretics, while current techniques for TRALI treatment are supportive. The risk of VAP can be reduced through hospital-wide practices promoting patient and provider hygiene, along with specific maneuvers like raising the head of the bed to prevent aspiration.^22,23^ Patients with VAP and/or ARDS should be assessed daily for extubation with spontaneous breathing trials. If severe ARDS demonstrates no improvement with the various positive pressure techniques, venovenous extracorporeal membrane oxygenation should be discussed.^24,25^

Abdominal compartment syndrome is a serious complication detected in patients who are difficult to ventilate and have sustained intra-abdominal pressure greater than 20 mm Hg while paralyzed. This complication can be avoided with preemptive diuretics and assessment for hemorrhage and can be managed with decompression laparotomy, diuretics, dialysis/hemofiltration, and intravenous paralytic agents. Patients with ischemic colitis present with abdominal distension and/or pain, fevers, early bowel movements (post-op day 0/1), leukocytosis, and persistent acidosis. Early diagnosis with Flex Sig is crucial with this complication, which may require intraoperative revascularization of the IMA or diseased SMA for prevention. Treatment involves an exploratory laparotomy with possible bowel resection and supportive care.^4^

Acute kidney injury (AKI) is a renal complication avoidable by minimizing vasopressors, avoiding prolonged suprarenal aortic cross-clamping, and avoiding nephrotoxic drugs. AKI is diagnosed in patients with FENa greater than 1%, muddy brown casts in urine sediment, low urine output, and anasarca. Renal replacement therapies (ie, dialysis) may be necessary for treatment of AKI.^4^

## Post-Discharge

### Late Complications to Monitor/Manage

**Figure 2:**
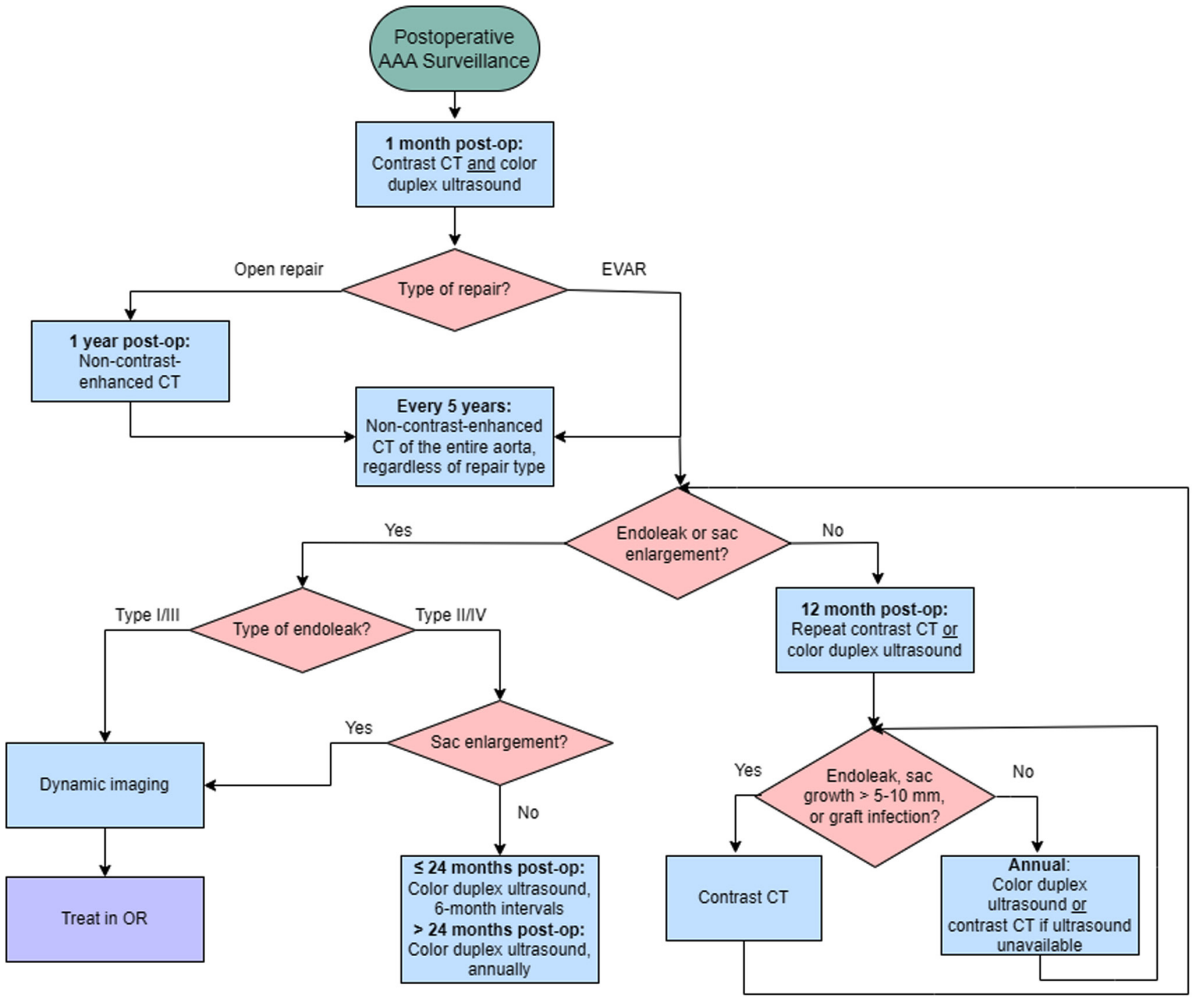
Algorithm for postoperative ruptured aortic abdominal aneurysm surveillance based on Society of Vascular Surgery guidelines and Houston Methodist Hospital protocol.^2^ CT: computed tomography; EVAR: endovascular aortic repair; OR: operating room.

Despite the less invasive nature of EVAR lending to its lower mortality and lower index of length of stay, greater readmission numbers have been reported for EVAR than OSR after RAAA treatment.^26^ The highest median 30- and 90-day readmission costs are associated with endoleaks, a unique complication of EVAR that necessitates careful postoperative surveillance with contrast CT imaging and color duplex ultrasound (Figure 2).^1,27^ To avoid endoleaks that require urgent treatment (type I and III), EVAR stent grafts should be oversized since measurements of vessels from CT images in patients who are typically hypotensive and/or unstable are smaller than normal due to vasoconstriction and shock.

Other late complications include device migration and aortic graft infection. Device migration occurs if EVAR is done outside of the instructions for use, especially if there is an inappropriate proximal sealing zone causing the graft to drop into the aneurysm over time. Aortic graft infection affects both EVAR and OSR patients and may arise as a late complication. Patients may present with generalized sepsis, groin drainage, pseudoaneurysm formation, and ill-defined pain. Preventative measures include antibiotic prophylaxis, and treatment involves graft excision and OSR followed by antibiotic therapy.

### Patient Quality of Life

As the healthcare model and research objectives shift towards improving patient outcomes, assessment of patient quality of life post-discharge is a fundamental step in a systems-based approach to creating center-specific policies that address recurrent issues reported by patients. To gauge the impact of the hospital visit and RAAA treatment on the patient’s daily life, we recommend using the EQ-5D-5L survey, which analyzes five dimensions of health: mobility, self-care, usual activities, pain/discomfort, and anxiety/depression.^28^ The survey can be distributed via email or through a patient portal. This quality-of-life surveillance can be used to improve patient outcomes through the help of social workers and case management in the outpatient setting.

## Conclusion

Management of acute aortic conditions such as RAAA requires clear, timely communication between the various members on the medical team as the patient is transferred throughout the different phases of surgical care. We describe the current RAAA protocol at the HMH AATC to educate future vascular surgeons and serve as recommendations for low-volume aortic centers. Assessment of hemodynamic stability is critical from the moment of patient transfer to discharge, and permissive hypotension should guide fluid resuscitation. EVBC is recommended in patients who are hemodynamically unstable, followed by EVAR or OSR. The intraoperative and postoperative complications discussed should be prevented, if possible; however, early recognition and treatment in the OR or ICU may help lower the risks of morbidity, mortality, and long-term complications.

## Key Points

- HIPAA-compliant cloud-based image reviewing systems should be available in centers treating acute aortic syndromes to assess the patient disposition and determine whether the patient should go to the intensive care unit (ICU) or operating room (OR) upon arrival, as well as the type of operation required to treat the acute aortic syndrome.
- Patients with acute aortic syndrome who require surgical intervention should go directly to the hybrid OR, where all techniques are available to treat the condition. Ruptured abdominal aortic aneurysm (RAAA) Timeout must include the type and time of pressor, crystalloid, colloid, and blood product administration as well as the evaluation of patient consciousness level by the vascular surgeon.
- Permissive hypotension and the patient’s level of consciousness should guide preoperative fluid resuscitation to avoid major blood loss and postoperative complications associated with volume overload and transfusion.
- We recommend endovascular balloon control (EVBC) in hemodynamically unstable patients regardless of the operative plan, be it endovascular repair (EVAR) or open surgical repair.
- The patient with RAAA should be treated with EVAR under local anesthesia with or without proximal EVBC, when possible. Surgeons should avoid EVAR for RAAA outside of instructions for use.
- Early recognition of postoperative complications in RAAA patients can guide early and appropriate management in the ICU or in the OR.
